# Bioavailability of Microencapsulated Iron from Fortified Bread Assessed Using Piglet Model

**DOI:** 10.3390/nu9030272

**Published:** 2017-03-13

**Authors:** Malgorzata A. Bryszewska, Luca Laghi, Augusta Zannoni, Andrea Gianotti, Francesca Barone, Danielle L. Taneyo Saa, Maria L. Bacci, Domenico Ventrella, Monica Forni

**Affiliations:** 1 Faculty of Biotechnology and Food Sciences, Lodz University of Technology, Lodz 90-924, Poland; malgorzata.bryszewska@p.lodz.pl; 2 Department of Agro-Food Science and Technology, University of Bologna, Cesena 47521, Italy; l.laghi@unibo.it (L.L.); andrea.gianotti@unibo.it (A.G.); danielle.taneyosaa2@unibo.it (D.L.T.S.); 3 Department of Veterinary Medical Sciences, University of Bologna, Ozzano dell’Emilia 40064, Italy; augusta.zannoni@unibo.it (A.Z.); francesca.barone7@unibo.it (F.B.); marialaura.bacci@unibo.it (M.L.B.); monica.forni@unibo.it (M.F.)

**Keywords:** microencapsulated iron, fortified bread, iron bioavailability, piglet model, iron deficiency, anemia, metabolome

## Abstract

The aim of this study was to investigate the influence of oral iron supplementation, in the form of fortified breads, on the growth performance, health, iron status parameters, and fecal metabolome of anemic piglets. A study was conducted on 24 hybrid (Large White × Landrace × Duroc) piglets. From day 44, the post-natal 12 piglets were supplemented with 100 g of one of two experimental breads, each fortified with 21 mg of ferrous sulphate, either encapsulated or not. After one week of oral supplementation, hematological parameters (hematocrit value, hemoglobin, and red blood cells) showed statistically significant differences (*p* ≤ 0.05). Piglets fed with the fortified breads had higher iron concentrations in the heart, liver, and intestinal mucosa compared to anemic piglets fed with control bread. Gene expression of hepcidin, iron exporter ferroportin (IREG1), and divalent metal transporter 1 (DMT1), together with concentrations of plasma ferritin, showed no significant statistical differences between groups. Both fortified breads could be used as sources of bioavailable iron. The seven-day intervention trial showed microencapsulation to have only a mild effect on the effectiveness of iron supplementation in the form of fortified bread.

## 1. Introduction

Iron is an essential metalloelement for the physiology and biochemistry of most forms of living organism [1]. Well-nourished people generally have 4 to 5 grams of iron in their bodies, making it the most abundant trace element in the human body [2,3]. The functional iron pool consists of structural components in heme proteins—hemoglobin, myoglobin, and cytochrome-dependent proteins [3]. Approximately 2 grams of iron is stored by adult men in proteins such as ferritin and hemosiderin, and somewhat less by women of childbearing age. Both the functional and stored iron pools are maintained by the balance between intestinal absorption of dietary iron and iron losses via the gastrointestinal tract, loss of blood (e.g., menstruation), sweat, skin, or urine [4,5]. 

The absorption of dietary iron is regulated by physiological factors such as bioactive hepcidin peptide, iron importer divalent metal transporter 1 (DMT1), and the cellular iron exporter ferroportin (IREG1) [6]. The molecular target of hepcidin is IREG1, which supplies iron to plasma from duodenal enterocytes engaged in dietary iron adsorption, from macrophages of the spleen and liver which recycle old red blood cells, and from hepatocytes involved in iron storage [7,8]. The interaction of hepcidin and IREG1 results in the internalization and degradation of the ligand-receptor complex, which decreases the delivery of iron to plasma. DMT1 is the most important apical uptake transporter of inorganic iron in enterocytes, which import ferrous iron. DMT1 is essential for normal iron homeostasis [6].

Iron deficiency is the most common nutritional disorder, affecting people of all ages worldwide, and mammals in general [9,10]. According to the WHO, nutritional iron deficiency affects 1.5–2 billion people worldwide [11,12]. Iron deficiency is associated with diminished work productivity, lower immunity, and impaired cognitive development. Iron deficiency can be caused by inadequate iron intake, compromised bioavailability, and increased iron losses, or a combination of these. The predominant dietary form of iron is non-heme iron, which accounts for 70%–90% of dietary iron intake in the world, with higher intake in the developing world than in developed countries [13]. Non-heme iron absorption is in the range of 5%–15%. The two major dietary inhibitors of absorption are phytates and polyphenols [14]. The most significant enhancer of iron bioavailability is ascorbic acid, which both reduces and chelates iron, rendering it soluble and available for absorption in the gut [15]. 

Iron deficiency can be treated using oral iron supplements, such as ferrous sulfate, ferrous gluconate, and ferrous fumarate [16]. However, inorganic iron supplements have limitations, including poor bioavailability and potential adverse effects in the gastrointestinal tract, primarily due to the oxidative toxicity of ferrous iron [17]. New types of iron supplements with minimal side effects and dietary sources with high absorption efficiency, such as fortified foods, are therefore needed. Food fortification is considered the most cost-effective population-based strategy in the long-term to combat micronutrient malnutrition, effective with no or fewer side effects than supplementation [18].

This study set out to investigate the effects of two experimental breads—each fortified with ferrous sulphate, either encapsulated or not—on iron status indicators, with piglets used as models. Iron encapsulation was chosen because of its potential to help overcome unwanted sensory changes in fortified food, reduce interactions of Fe with food components, and increase Fe bioavailability [19,20]. Swine provide possibly the best preclinical animal model for investigating the gastrointestinal system and its functions [21,22]. New piglet models are constantly emerging [23]. Iron deficiency is extremely common in pigs, and is considered to be a para-physiological condition by zootechnical standards. Indeed, it is mandatory to supplement piglets with exogenous iron within few days of birth, in order to compensate for poor iron storage by the mothers and the immaturity of the piglets’ absorption mechanisms [24]. This species is therefore extremely useful for use in preclinical trials concerning iron deficiency and food supplementation.

## 2. Material and Methods

### 2.1. Experimental Breads and Diet of Piglets

The control bread (CB) and iron fortified breads (FeMB and FeSB) were made according to a procedure developed and optimized at the University of Bologna (UNIBO). The breadmaking process was based on dried compressed yeast (Lesaffre, San Quirico, Italy). The dough was fermented for 45 min at a controlled temperature (30 °C) and then portioned. The pieces were fermented for another 45 min under the same conditions, then baked at 195 °C for 45 min. All samples were obtained from the same batch.

The fortified bread was made from an iron fortified mix of white wheat flour (type 650) and encapsulated ferrous sulphate supplied by EPSA (Valencia, Spain). The experimental breads were produced in the form of sandwich loafs, sliced, and fresh frozen. Each morning, a small aliquot was thawed and given to the animals after crumbling.

Analysis of the humidity, fat content, and content of soluble sucrose/saccharose in the breads was performed according to Polish standard norm ref. no PN-A-74108:1996, Bakery products—testing methods. Protein content was calculated based on nitrogen content, determined using the Kjeldahl method (AOAC 950.36-1950, Protein in bread). The ash residue of the flour was measured using AOAC Official method 923.03. Total dietary fiber was quantified using an assay kit (Sigma, St. Louis, MO, USA). 

A low iron swine diet (LISD), providing the lowest possible amount of iron, was designed for the purposes of the experiment. The standard commercial swine feed could not be used as the basal diet, due to its iron content equivalent to dietary requirements. The LISD was composed of corn flour (40%), barley flour (10%), barley flakes (20%), soybean flour (20%), and bran (10%). The iron content .in this diet formula was one tenth of that found in standard commercial swine feed. 

### 2.2. Animals

Twenty-four commercial hybrid (Large White × Landrace × Duroc) piglets, born within the ASA Unit (DIMEVET, UNIBO), were enrolled in the study. Twelve of the animals were born of one sow, and the other 12 to two different sows 4 days later. All of the sows were inseminated using artificial insemination doses from the same boar, in an attempt to standardize the piglets as much as possible. The animals were monitored daily to assess their clinical condition. All of the activities performed on the animals were approved by the Local Ethics Committee and the Italian Ministry of Health (Legislative Decree 116/92, protocol number 10-75-2013).

### 2.3. Trial Design and Feeding Protocol

Five days after birth (P5), the animals were randomly assigned to four groups (six piglets in each group, *n* = 6). Only one of the four groups received regular intramuscular (IM) supplementation with 1 mL iron dextran (Endofer, Fatro IT, 100 mg·iron·mL^−1^). Groups are represented in Table 1. The experiment was divided into two identical blocks to guarantee better management of the animals and to ensure that they were of the same age at the starting point. During their first 14 days of life, the piglets were suckled on sow milk only. During the last phase of lactation (P14), LISD was placed in the mangers of the farrowing crates to accustom the animals to solid feed. The same diet was then left in quantities sufficient for the piglets to eat ad libitum throughout the rest of the trial.

In order to start training the piglets to eat the experimental breads, control bread (CB) was added to the LISD until weaning. After weaning (P28), the piglets were individually fed with 50 g of CB each day. Starting on day 44 (P44), the animals were individually fed 100 g of either the control or fortified bread, depending on the experimental group, for 7 days until the end of the trial (P51). 

Two groups were fed with iron fortified experimental breads. One group was fed microencapsulated iron bread (FeMB-G1), the other was fed bread containing the same quantity of iron sulphate, starch and citric acid (FeSB-G2). The remaining two groups were the control groups. One (CB-G3) ate control bread, while the other (CB-G4) ate control bread and was given IM iron supplements from P5. 

To guarantee that the piglets ingested the required quantity of bread, it was mixed with water (100 g of bread, 130 mL^−1^ water) and fed to the piglets through a 50 mL syringe connected to a silicon tube, which the animals were given to suck. Vanilla extract was added to improve the flavor. The weight of individual piglets was monitored periodically (P5, P28, P44, and P51) using a livestock scale (CO.BA di Melloni Giuseppe e C. SNC, Pieve di Cento IT).

### 2.4. Sampling Protocol

Blood samples were collected at P5, P44, and P51. Fecal samples were collected from the rectum at P44 and P51. Urine and tissue samples were collected only at P51, upon euthanasia.

Blood samples were collected under general anesthesia via the femoral artery, using a 21G butterfly needle and a vacuum system. Tubes with K3EDTA anticoagulant and clot activator were used (Venosafe^®^, Terumo, Leuven, Belgium). Samples in K3EDTA for a complete blood count (CBC), and serum (obtained by 1000 *g* × 10 min centrifugation) for biochemical profiling, were analyzed by the Veterinary Clinical Pathology Service of DIMEVET (UNIBO).

At the end of the trial, the animals were sedated by an intramuscular (IM) injection of Tiletamine-Zolazepam (Zoletil, Virbac, Praga) (5 mg·kg^−1^). General anesthesia was induced 10 min later, using an intravenous (IV) bolus of Thiopental sodium (Pentothal, MSD Animal Health s.r.l.) (10 mg·kg^−1^). After blood and urine had been collected, the animals were euthanized by an IV bolus of Tanax (0.3 mL·kg^−1^, Intervet, Milano, Italy) for extensive sampling.

Immediately after euthanasia, the digestive tract was removed and separated into its anatomical parts. Mucosal scraping of the stomach wall (lower body part, near pyloric antrum) was performed using a glass microscope slide, and the mucosa was flash frozen in liquid nitrogen. A 25 cm long segment of the small intestine (beginning ~10 cm distal to the pyloric sphincter) was removed, cut open longitudinally, and rinsed with PBS [25]. Duodenal scrapings from the first 10 cm of exposed mucosa were taken and the tissue was immediately frozen in liquid nitrogen for gene expression analysis. The second 15 cm of mucosa was scraped and frozen for total iron content analysis. Samples of organs (liver, heart, spleen, and kidney) were in part conserved for gene expression analysis using RNAlater (Ambion, ThermoFisher, Waltham, MA, USA) and in part flash frozen in liquid nitrogen for iron determination. Stomach contents, feces, and urine were collected in sterile tubes, frozen immediately, and stored at −20 °C.

### 2.5. Hematological Tests

CBC was performed using an automated hematology analyzer (ADVIA 2120, Siemens Healthcare Diagnostics, Tarrytown, NY, USA). All of the classic hematological variables were analyzed, along with relatively new platelet and reticulocyte indices.

Biochemical analyses were performed on an automated chemistry analyzer (Olympus AU 400, Beckman Coulter/Olympus, Brea, CA, USA), including analyses of total iron (TI) and unsaturated iron binding capacity (UIBC). TI and UIBC, analyzed using a colorimetric method, were used to further calculate the total iron binding capacity (TIBC) and the TIBC saturation percentage (TSAT). 

### 2.6. Total Iron and Fe(II)

The concentration of iron in wet-digested samples of organs (liver, spleen, heart, and kidney), whole blood, diets, and feces was determined using atomic absorption spectrophotometry. The Standard Berghof (Berghof Products + Instruments GmbH Labor Technik, Eningen, Germany) method for microwave digestion of wheat was applied to the gastric content and whole blood samples. Briefly, 1 g or 0.5 mL (blood) samples were digested in 10 mL of concentrated nitric acid and 2 mL hydrogen peroxide using a three-step digestion program, with the temperature raised to 170 °C and a total digestion time of 25 min. For the intestinal mucosa samples, the volume of acid and hydrogen peroxide was reduced by half. Before digestion, the samples of gastric content were homogenized using a Glas Col GKH Control Stirrer Mixer.

The digested samples were diluted with deionized water and the iron content was determined by AAS using a GBC 932 spectrophotometer (GBC Scientific Equipment Pty Ltd., Braeside, Australia), with a hollow cathode lamp for iron, at 248.3 nm. Acetylene and air flow were fine-tuned daily. Standard curves were prepared daily by diluting iron standard reference materials (1000 µg·cm^−3^; JT Baker*^®^*Chemicals, Phillipsburg, NJ, USA) with deionized water to concentrations of between 0 and 10 µg·cm^−3^. Certified reference material, whole blood L-1 (Seronorm™ Trace Elements Serum L-1), was used to test the accuracy of the methods. Iron recovery from whole blood was 97%. During analysis, precautions were taken to avoid contamination of the iron samples. A ceramic cutter was used. The glassware was washed in 10% HCl and rinsed three times in ultrapure water. 

The presence of Fe(II) was determined through the reaction of formation blue coordinating compound, formed by chelation of Fe(II) with Ferene S reagent (3-(2-pyridyl)-5,6-difurylsulfonic acid-1,2,4-triazine sodium salt) under acidic conditions [26].

### 2.7. RNA Isolation and Quantitative Real Time PCR (qPCR) for HAMP, IREG1, and DMT1

Total RNA was isolated from liver and duodenal mucosa samples using a NucleoSpin^®^RNA Kit (Macherey-Nagel GmbH & Co. KG, Düren, Germany). One microgram of total high quality RNA (A260/A280 ratio above 2.0) was reverse-transcribed in cDNA using an iScript cDNA Synthesis Kit (Bio-Rad Laboratories Inc., Hercules, CA, USA), with a final volume of 20 μL. Real-time quantitative PCR was carried out using a CFX 96 Real Time System (Bio-Rad) and iTaq Universal SYBR Green Supermix (Bio-Rad). All samples were analyzed in duplicate (10 µL/well). The amplification reaction (20 µL) contained 10 µL of iTaq Universal SYBR Green Supermix, 0.8 µL of each forward and reverse primer (5 µM), and 2 µL of cDNA. All samples were performed in duplicate. Controls lacking a cDNA template were included to determine the specificity of target amplification. The real-time program included an initial denaturation period of 1 min 30 s at 95 °C, 40 cycles at 95 °C for 15 s, and 60 °C for 30 s, followed by a melting step with ramping from 55 °C to 95 °C at a rate of 0.5 °C/10 s. The specificity of the amplified PCR products was confirmed by melting curve analysis and agarose gel electrophoresis. The expression of the hepcidin gene (HAMP, hepcidin antimicrobial peptide) was evaluated on cDNA derived from liver tissue, while the gene expression of iron transporters (IREG1 and DMT1) was assessed on cDNA derived from duodenum. The swine primer sequences are reported in Table 2. 

The relative expressions of the studied genes were normalized based on the geometric mean of two reference genes (GAPDH, glyceraldehyde-3-phosphate dehydrogenase and RPL35 ribosomal protein L35). The relative mRNA expression of the tested genes was evaluated in relation to the control group (CB-G4) using the 2^−ΔΔ*C*t^ method [27].

### 2.8. Ferritin in Plasma

The specific Pig Ferritin Sandwich ELISA (Cod. LS-F6483, LifeSpan BioScience, Inc., Seattle, WA, USA) was used for the ferritin assay. Plasma ferritin concentrations were extrapolated from a standard curve, as described in the manufacturer’s protocol.

### 2.9. Metabolomics of Feces

Fecal samples were prepared for proton nuclear magnetic resonance spectroscopy (^1^H-NMR) analysis by vortex mixing 80 mg of stool for 5 min with 1 mL of deionized water, followed by centrifugation, for 15 min at 18,630 g and 4 °C. Seven hundred microliters of supernatant was added to 100 μL of a 10 mM solution of 3-(trimethylsilyl)-propionic-2,2,3,3-d4 acid sodium salt (TSP) in D_2_O, buffered at pH 7.00 with 1 M phosphate buffer. Immediately before analysis, the samples were centrifuged again. ^1^H-NMR spectra were recorded at 298 K using an AVANCE III spectrometer (Bruker, Milan, Italy) operating at 600.13 MHz.

The HOD residual signal was suppressed by applying the first increment of the NOESY pulse sequence and a spoil gradient [30]. The NOESYGPPR1D sequence, part of the standard pulse sequence library, was used. Each spectrum was acquired by summing up 256 transients using 32 K data points over 7211.54 Hz (for an acquisition time of 2.27 s). To apply NMR as a quantitative technique, the recycle delay was set to 5 s, taking into consideration the longitudinal relaxation time of the protons [31]. The signals were assigned by comparing their chemical shift and multiplicity with the Human Metabolome Database and Chenomx software data bank (Chenomx Inc., Edmonton, AB, Canada, ver 8.1) [32].

The ^1^H-NMR spectra were pre-processed for quantitative analysis following a method already described in literature [30]. Briefly, the spectra were baseline-adjusted by means of simultaneous peak detection and a baseline correction algorithm (SPDBC) implemented in the baseline R package [33]. The spectra were corrected for errors in chemical shift misalignments using an in-house modified version of Correlation Optimized Shifting (i-Coshift) [34].

### 2.10. Statistical Data Analysis

The data were analyzed using a Student’s *t*-test and one-way analysis of variance (ANOVA), followed by a Tukey post hoc comparison test (SPSS program version 13.0; SPSS Inc., Chicago, IL, USA). Differences of at least *p* ≤ 0.05 were considered significant. Iron concentration underwent statistical analysis in R computational language. In each group, data were considered as probable outliers when they exceeded by 1.5 times the 0.25–0.75 percentile range. Metabolomics data were analyzed in R, with sparse principal component analysis (sPCA) performed using a mixOmics package, applying the sparsity principle as detailed by Shen and Huang [35].

## 3. Results and Discussion

### 3.1. Experimental Bread

Three types of experimental bread were prepared, using conventional yeast fermentation: microencapsulated iron fortified bread (FeMB), ferrous sulphate fortified bread (FeSB), and control bread (CB) without any iron supplementation. The formula of the FeSB was based on that of FeMB, but instead of microencapsulated iron an equivalent amount of modified starch, ascorbic acid, and ferrous sulphate was used, in the form of individual compounds. The content of iron in the fortified breads was 21 mg Fe 100 g^−1^·WM (Wet Mass), ten times higher than that in the control bread. To meet WHO recommendations for cereal flour fortification, ferrous sulphate was selected as the iron source [36]. The presence of ferrous ions in the final experimental breads was determined, as ferrous ions are easily oxidized to ferric ions, especially under the conditions used in the baking process (high temperature and humidity), which may accelerate the reaction. The presence of Fe(II) in the experimental breads was confirmed in a reaction with Ferene S. During the trial period, the daily iron intake of each piglet was 21 mg. This dose complies with experimental findings and recommendations. According to the National Research Council’s current guidelines, neonatal pigs require 7 to 16 mg of iron per day for normal growth [37]. Table 3 presents the composition of the experimental breads.

### 3.2. Body Weight and Blood Analyses

The body weights of the piglets and their main blood parameters are reported in Table 4. 

Parameters showing statistical differences and their correlations are reported in the upper part of the table. All piglets were of similar weight on day 5 (data not shown) and analysis showed that all of the animals enrolled in the study were healthy. The group that received IM iron supplements subsequently gained significantly more weight than those which did not receive the experimental diet. This might be explained by the fact that animals that received the IM supplementation at 5 days, had more time (from P5 to P44) to restore and correct anemia and balance all of the physiological gastrointestinal mechanisms. Their weight is indeed significantly higher since the beginning of the trial P44. Over the week of the trial, the differences were maintained between the live body weights of the piglets in the positive control group (CB-G4) and those of the piglets in all other groups.

Episodes of diarrhea were reported from the second day of the experimental diet. However, none of the four groups showed a higher incidence of diarrhea than the others. Thomaz et al. [38] report that animals fed diets supplemented with trace minerals in inorganic form suffer higher rates of diarrhea than animals fed diets without mineral supplements. However, this was not confirmed in the present study.

Levels of variables such as hematocrit (HCT), hemoglobin (Hb), and red blood cells (RBC) seem to have been influenced by the kind of bread administered (Table 4). Groups that received iron fortified breads (FeMB-G1; FeSB-G2) showed significant increases in these parameters (*p* ≤ 0.05) as compared to piglets fed with the control bread (CB-G3 and CB-G4). At the beginning of the experiment, the FeSB-G2 group was classified as anemic, whereas after the treatment they showed only borderline anemia [39]. The impact of iron microencapsulation on Hb was not as marked as that of unencapsulated ferrous sulfate, although there was no significant difference between the treatments (*p* > 0.05). 

No changes in levels of leukocytes were observed in any of the groups over the course of the experiment. Clinical chemistry analyses did not reveal significant variations at different times or between groups, proving that the animals were healthy. CB-G4 showed the highest concentrations of Gamma-glutamyl transferase (GGT) at P44. By the end of the trial, GGT had increased in the groups fed with iron fortified breads (FeMB-G1 and FeSB-G2), reaching or exceeding the level observed previously for CB-G4, while it remained stable in group CB-G3 (the lowest noted concentration). Therefore, this parameter seems to be directly related to iron supplementation, since it increased after both IM and oral administration. In all of the groups, the glucose concentration in the blood was higher on day 51 than on day 44. This is related to the fact that the animals were fed 1 hour before euthanasia in order to collect gastric content. The iron content in serum varied widely between animals. Despite this large individual variation, it can be noted that the iron levels in orally supplemented groups (FeMB-G1 and FeSB-G2) decreased, while they remained stable in groups fed with control bread (CB-G3 and CB-G4). Our hypothesis is that the increase in nutritional iron availability may stimulate iron storage or tissue-binding mechanisms, therefore lowering the UIBC (Unsaturated Iron Binding Capacity) and TIBC (Total Iron Binding Capacity). TSAT (TIBC Saturation Percentage) appears to have remained stable, with minimal variations. 

CB-G4 did not show large differences for the main indices of hematopoiesis and anemia. The probable reason is that the diet used (LISD) was designed to have a low iron content. Whereas, a normal farm practice is IM supplementation and the use of specific iron-rich diets. 

Overall, all of the blood parameters fell within the physiological reference intervals available in the literature for piglets. Therefore, despite some differences between the groups at P44 and P51, the trial did not determine any dangerous or important alterations in the animals, which remained healthy throughout the trial [40]. 

### 3.3. Relative Expression of HAMP, IREG1, and DMT1

**Figure 1:**
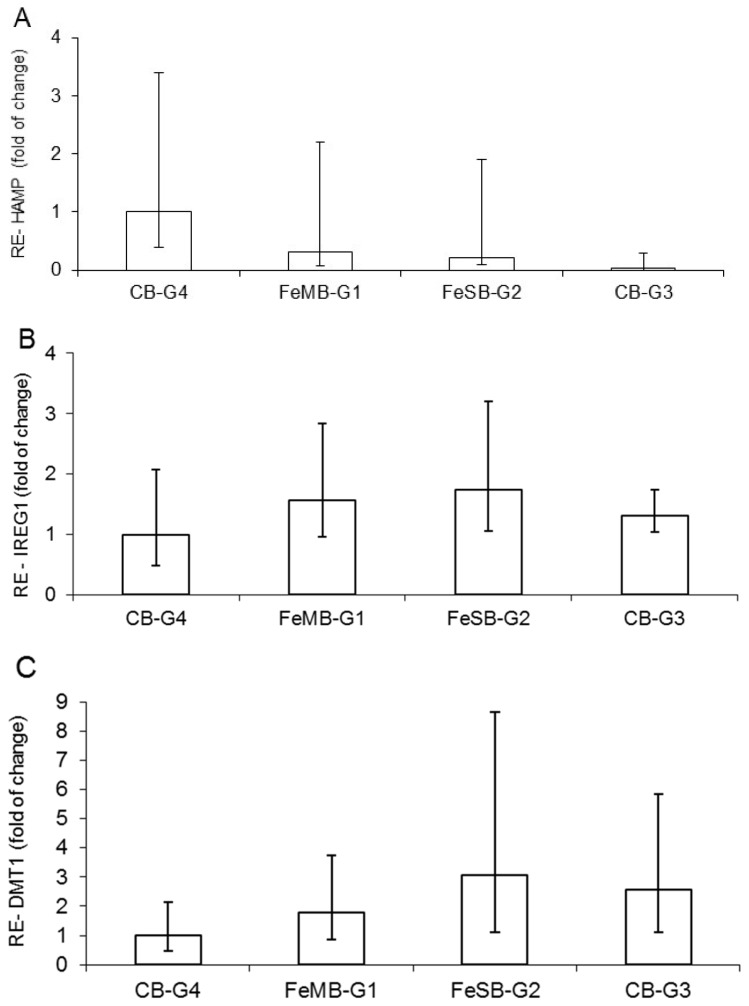
Effect of different breads on relative expression (RE) of hepcidin (HAMP) mRNA in liver (**A**); ferroportin (IREG1) (**B**); and Fe importer divalent metal transporter 1 (DMT1) mRNA in duodenum (**C**) determined by qPCR. Data are expressed as fold changes with respect to CB-G4. FeMB-G1: no iron supplementation/Microencapsulated Fe Fortified bread; FeSB-G2: no iron supplementation/Free Fe Fortified bread; CB-G3: no iron supplementation/control bread; CB-G4: iron supplementation/control bread.

The effects of the different bread types on mRNA of hepcidin (HAMP) in the liver, and on IREG1 and DMT1 at the duodenal level, did not differ in a statistically significant manner (Figure 1). Moreover, the gene expression of HAMP calculated with reference to CB-G4 shows a progressively decreasing trend, starting from the group of anemic piglets fed bread with microencapsulated iron (FeMB-G1), followed by the group given non-encapsulated iron bread FeSB-G2 and finally the anemic group, CB-G3, which was fed with control bread (Figure 1A). This trend, showing a slight up-regulation in groups with iron fortified bread in respect to the anemic group, can be explained by the fact that iron is the most important proven biological factor for inducing hepcidin expression, while there is also evidence that both iron saturation of plasma and hepatic iron loading stimulate hepcidin synthesis [25,29].

The relative gene expression of IREG1 (Figure 1B) and DMT1 (Figure 1C) in relation to the group with encapsulated iron supplementation (CB-G4) showed a relatively higher value for piglets in FeSB-G2. The increasing trend in intestinal DMT1 mRNA (Figure 1C) for piglets fed with FeMB-G1 and FeSB-G2 is likely due to the animals’ anemic status and increased need for dietary Fe to meet erythropoietic demands. The increasing trend in IREG1 expression observed for animals fed with FeMB-G1 and FeSB-G2 as compared with CB-G4 corresponds to the opposite trend observed for HAMP expression. Hepcidin binds to IREG1, causing internalization and degradation of the protein and decreasing cellular Fe export [7,41].

### 3.4. Ferritin Determination

Although ferritin is mainly found as a cytosolic protein, small amounts of ferritin are secreted into serum, in amounts closely reflecting the size of the body’s iron stores [42,43]. Plasmatic ferritin can be used to identify differences within the physiological range, reflecting non-heme iron levels in the liver and spleen more than other blood values [44].

**Figure 2:**
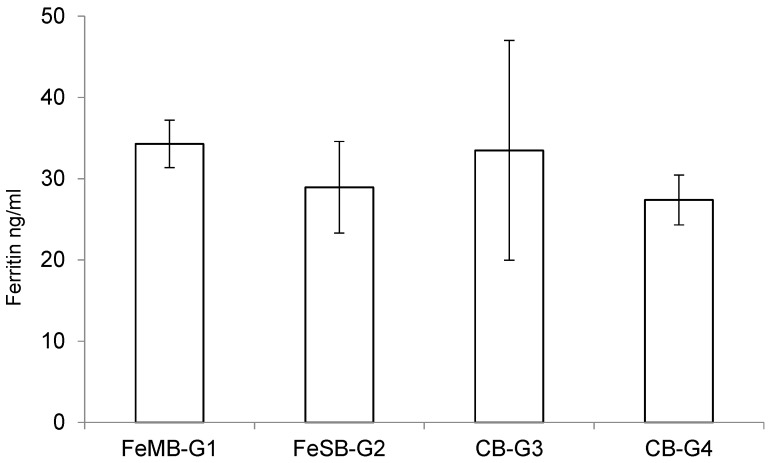
Effect of consuming different breads on plasmatic levels of ferritin in piglets.

The effects of consuming the different types of bread on the piglets’ plasmatic levels of ferritin are presented in Figure 2. There were no statistically significant differences between the groups in terms of the amounts of ferritin detected in the plasma samples using a specific ELISA kit, although the group fed with FeMB-G1 had the highest ferritin levels. This lack of significant differences may be due to the short duration of the experiment.

### 3.5. Iron Absorption and Storage

The total iron content in the intestinal mucosa and internal organs after 51 days of life is shown in Table 5. The iron content in the intestines of the animals which did not receive iron supplementation was 18.4 ± 4.6 mg·Fe·kg^−1^. This was significantly lower (*p* < 0.05) than in CB-G4. In contrast, the iron content in the intestinal mucosa of the animals that received either oral or IM iron supplementation ranged from 25.0 to 28.0 (mg/kg·WM), and did not differ from the CB-G4 group (*p* > 0.05). Iron content in the intestinal mucosa is known to be controlled tightly by double regulation [3]. The first regulatory mechanism controls the passage of iron into intestinal mucosal absorptive cells (enterocytes). Iron transfer from the basal surface into the blood is then regulated according to the body’s needs at the time. Excess iron entering the mucosal cells can be incorporated into ferritin, stored in the intestinal cells for 2–3 days, and finally lost by exfoliation and apoptosis of the cell [37]. The iron levels of the piglets fed with iron fortified breads were close to iron adequate status. This suggests that iron absorbed from lumen was not stored in the epithelial cell, but uptaken by transferrin and transported systemically to the body. 

Oral supplementation with iron in FeMB or FeSB forms resulted in a statistically significant increase in the iron content of the liver, by up to 51.6% and 53.9%, respectively, with respect to the CB-G3 group. The concentration of Fe showed a clear tendency to increase in orally supplemented groups compared to CB-G3, confirming the positive effect of this Fe source on absorption and storage in the body.

To understand the extent to which oral supplementation moved iron content away from typical levels for anemic non-supplemented piglets, we calculated the differences shown by each supplemented piglet from those of the piglets in groups CB-G3 and CB-G4. We then set up a paired comparison between the distances, using Welch’s *t*-test. [45] The supplemented piglets, independent of the kind of supplementation, showed an intermediate iron content in relation to the CB-G3 and CB-G4 groups in each of the studied body organs with the exception of liver, where the iron content was closer to that of CB-G3.

Piglets given increased daily iron intake in the form of FeMB-1 or FeSB-2 showed significantly higher iron content in their hearts in comparison with CB-G3 (*p* < 0.05), but did not reach the level observed in piglets given IM supplementation from day 5 of life. In both orally supplemented groups, iron accumulation in the heart and liver, the organs most commonly affected by iron overload, remained below the values observed in CB-G4. At the end of the intervention trial, there were no statistical differences in terms of iron content in the spleens of supplemented animals, where iron content reached similar levels of around 10 mg·g^−1^. The iron content in the spleens of animals not given supplementation was lower, although the increase caused by one week of oral supplementation was not statistically significant. 

A comparison of the iron content in feces from individual animals showed a broad range of variation. In the feces of the FeMB-1 group, iron content varied from 94 to 180 mg/kg on 44 day and from 282 to 485 mg/kg on day 51. However, with increased iron intake there was an up to twofold increase in excretion. At the same time, in the CB-G3 iron excretion decreased by half.

Pigs fed with FeSO_4_ or microencapsulated FeSO_4_ had higher iron concentrations in the heart, liver, and intestinal mucosa compared with iron-deficient pigs, indicating that iron supplementation was effective at improving the iron status of the tested organs. These results are in agreement with those obtained by Fang et al., [46] who observed that 10 days of supplementation in the form of ferrous sulfate or iron glycine chelate increased iron content in the heart, liver, and lungs of iron-deficient piglets. 

### 3.6. Fecal Metabolome

**Figure 3:**
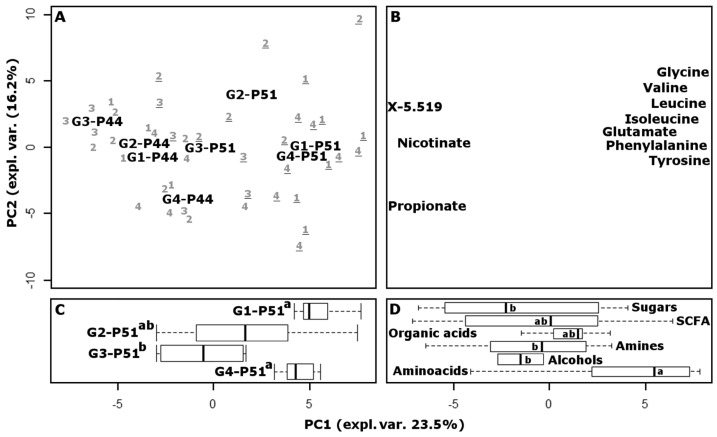
Score plot (**A**) of a PCA analysis of fecal metabolome. To facilitate the visualization of the differences of the samples at P51, their scores along PC1 are summarized by boxplots (**C**); Corresponding loading plot (**B**), in which only the 10 most important variables, as calculated by sPCA, are reported. The 81 loadings are also summarized by the molecular group in panel (**D**). In panels (**C**,**D**), different lowercase letters indicate statistically different groups (*p* < 0.05). (G1, G2, G3, G4 refer respectively to the groups fed with: microencapsulated iron bread (FeMB-G1); bread containing the same quantity of iron sulphate; starch and citric acid (FeSB-G2); control bread (CB-G3), and control bread with IM iron supplements at P5 (CB-G4)).

To provide an overview of the changes that occurred over the course of the experiment in the intestinal environments of piglets in different test groups, the metabolome of feces collected at the beginning and at the end of the experiment was investigated by means of high-resolution proton magnetic resonance spectroscopy (^1^H-NMR) and multivariate analysis. In several related studies, these methods have been found optimal for following the possible consequences of inflammation caused by exogenous agents [47,48,49]. Sixty-six molecules were quantified, mainly pertaining to amino acids, short chain fatty acids and organic acids. Fifteen other signals were detected, pertaining to different unknown molecules. The underlying structure of the data was represented in a multidimensional space using principal component analysis (Figure 3). The score plot highlighted the differences between the samples, while the loading plot provided a visual representation of the most important molecules that determined those differences. Along the first principal component (PC1), accounting for 23.5% of the total variance, the samples collected at P44 are grouped around negative values, while the samples collected at P51 are grouped around positive values. The linear combination of the molecules giving rise to PC1 therefore provides a superbly concise representation of the growth of the animals. Piglets belonging to group CB-G4 appear at P44 with less negative values than the others, which are closer to CB-G4 animals at P51, as a one tail Wilcox test confirmed. This was not unexpected, as such piglets were anticipated to be characterized by a more balanced physiological condition than those in any other group, with positive consequences for the overall development of the animals. The relative position of samples collected at P51 is remarkably different from the position of samples obtained at P44, as revealed by an ANOVA test. In fact, the CB-G4 samples are flanked by FeMB-G1, which has the highest PC1 values, by FeSB-G2 with intermediate values, and by CB-G3 which has the lowest values. 

A recent work on human subjects showed that some iron replacement therapies may cause inflammation accompanied by drastic modification of gut microbiota and metabolome [50]. Prompted by these findings, we investigated the intra-group distances of the piglets at P44 and P51, by considering the Euclidean space constituted by the concentration of all the molecules studied by NMR, scaled to unit variance. FeSB treatment caused a significant (*p* < 0.05) increase in the variability of the metabolome, with the average distance between FeSB-G2 piglets increasing by 175% after iron supplementation. This trend was confirmed when the samples were observed in the principal component space shown in Figure 3, or in the space of intermediate complexity. In contrast, groups FeMB-G1, CB-G3, and CB-G4 showed no statistical differences between P44 and P51, suggesting that iron supplementation through microcapsules had a milder effect on gut microbiota and metabolome than did fortification with iron sulphate. 

The loading plot of the PCA model reveals that the metabolic cycles mostly responsible for the highlighted trends involved the production of amino acids and decarboxylation. In fact, amino acids appear significantly more concentrated in samples located around positive PC1 values, while molecules belonging to the chemical group of amines appear more concentrated in samples with negative PC1 values.

## 4. Conclusions

This study has shown that bread fortified with ferrous sulphate or encapsulated ferrous sulphate provided effective treatment of anemia in piglets. Indeed, after a relatively short treatment time (7 days), animals initially classified as anemic, due to lack of exogenous iron supplementation, showed noticeable signs of improvement regarding hematological indicators of anemia. Fortified bread could therefore also provide a source of bioavailable iron for humans, which should be of interest to the functional foods industry. However, little difference was observed during the seven-day intervention trial between using microencapsulated iron or the same amount of ferrous sulphate. It might be expected that over the course of a longer study iron microencapsulation could obviate the negative effects of free iron supplementation, and this should be the focus of further research.

## Figures and Tables

**Table 1:** Experimental groups.

Treatments	Piglets Group			
	FeMB-G1	FeSB-G2	CB-G3	CB-G4
Intramuscular iron supplementation	No	No	No	Yes
Administered Bread	Microencapsulated Iron Bread	Non-Microencapsulated Iron Bread	Control Bread	Control Bread

**Table 2:** Primer sequences used for quantitative real-time polymerase chain reaction analysis.

Gene	Sequence (5′-3′)	PCR Product Length (bp)	Gene Bank Accession Number	Reference
HAMP	For: TCTCCCATCCCAGACAAGAC	123	NM_214117	Hansen et al., 2009 [25]
	Rev: AAGATGCAGATGGGGAAGTG			
IREG1	For: CTCTATGGAAACAGCCTTCTC	158	XM_003359590.1	Present study
	Rev: AGGATGACCGAAACATTCTG			
DMT1	For: AGGATCTAGGGCATGTGGTG	124	NM_001128440.1	Present study
	Rev: CCACAGTCCAGGAAGGACAT			
GAPDH	For: TGGTGAAGGTCGGAGTGAAC	120	AF017079	Dall’Aglio et al., 2011 [28]
	Rev: TGTAGTGGAGGTCAATGAAGGG			
RPL35	For: AACCAGACCCAGAAAGAGAAC	145	NM_214326	Alexander et al., 2012 [29]
	Rev: TTCCGCTGCTGCTTCTTG			

**Table 3:** Component analysis of breads administered to the pigs. All values are given in respect to the fresh bread mass. (FeMB: bread with microencapsulated iron; FeSB: bread with free iron; CB: control bread).

Sample/Breads	FeMB	FeSB	CB
Humidity (g 100 g^−1^)	38.90 ± 0.83	38.11 ± 0.19	41.27 ± 0.77
Proteins (g 100 g^−1^)	7.62 ± 0.53	8.10 ± 0.57	7.55 ± 0.53
Fat (g 100 g^−1^)	1.76 ± 0.20	1.43 ± 0.05	1.78 ± 0.42
Carbohydrates			
Total (g 100 g^−1^)	45.59	46.56	44.01
Soluble (Sucrose/Saccharose) (g 100 g^−1^)	1.35	1.00	0.69
Insoluble (fiber) (g 100 g^−1^)	1.61	1.61	1.61
Ash (g 100 g^−1^)	1.71 ± 0.04	1.73 ± 0.04	1.62 ± 0.05
NaCl (g 100 g^−1^)	1.47	1.47	1.47
Total Fe (mg 100 g^−1^)	21.10 ± 0.82	21.20 ± 0.63	2.80 ± 0.20

**Table 4:** Effect of bread administration on growth performance and blood parameters. Value are means ± SD. Means with different superscripts differ *p* < 0.05. Capital superscripts represent differences within the same group between P44 and P51. Lowercase superscript represent differences between the groups at the same experimental time. (BW: body weight; HCT: hematocrit; Hb: hemoglobin; RBC: red blood cells; WBC: white blood cells; RET: reticulocytes; GGT: gamma glutamyltransferase; TIBC: total iron binding capacity; UIBC: unsaturated iron binding capacity; TSAT: TIBC saturation percentage).

Intramuscular Iron Supplementation		Piglets Group			
		FeMB-G1	FeSB-G2	CB-G3	CB-G4
		no	no	no	yes
Body weight (kg)	P44	6.9 ± 1.33 ^a^	7.2 ± 1.5 ^a^	7,6 ± 1.0 ^a^	10.5 ± 1.5 ^b^
	P51	6.8 ± 1.1 ^a^	6.3 ± 1.4 ^a^	7.0 ± 1.6 ^a^	10.5 ± 1.9 ^b^
Hb (g/dL)	P44	6.1 ± 2.0 ^aA^	6.0 ± 2.5 ^aA^	5.3 ± 1.5 ^a^	8.8 ± 1.7 ^b^
	P51	7.2 ± 2.2 ^abB^	8.0 ± 2.5 ^abB^	6.3 ± 2.4 ^a^	9.3 ± 1.7 ^b^
HCT (%)	P44	21.7 ± 5.6 ^a^	21.1 ± 6.5 ^aA^	20.2 ± 4.8 ^a^	29.7 ± 3.6 ^b^
	P51	23.5 ± 5.5 ^ab^	25.5 ± 6.7 ^abB^	21.7 ± 5.9 ^a^	29.1 ± 4.1 ^b^
RBC (10^6^/mm^3^)	P44	5.10 ± 0.9 ^a^	4.59 ± 1.18 ^aA^	5.26 ± 1.68 ^a^	7.37 ± 0.86 ^b^
	P51	5.8 ± 0.9 ^a^	5.60 ± 1.12 ^aB^	5.78 ± 1.70 ^a^	7.27 ± 0.40 ^b^
WBC (10^3^/mm^3^)	P44	14.3 ± 2.2	11.5 ± 3.4	11.5 ± 1.6	17.5 ± 4.1
	P51	10.3 ± 3	8.1 ± 3.9	8.2 ± 2.5	14.1 ± 4.6
RET (10^3^/mm^3^)	P44	415.5 ± 189.2	634.1 ± 171.7	503.3 ± 64.5	299.1 ± 93.7
	P51	86.6 ± 57.9	112.4 ± 48.9	95.8 ± 28.5	58.6 ± 18.3
Glucose (mg/dL)	P44	107.3 ± 17.2	104.5 ± 9	112.5 ± 15.7	104 ± 7.3
	P51	162.2 ± 33.2	136.3 ± 26.9	150.8 ± 52.1	145.7 ± 27.3
GGT (U/L)	P44	32.5 ± 5.2	30.1 ± 3.5	28.3 ± 5.3	36.9 ± 4.2
	P51	38.4 ± 8.1	37.3 ± 8.3	29.3 ± 4.4	36.8 ± 5.5
Iron (serum) (µg/dL)	P44	97.2 ± 81.5	83.8 ± 59.1	41.8 ± 30.6	85 ± 47.2
	P51	66.3 ± 54.1	69.7 ± 47.8	39.8 ± 44	88.5 ± 42.5
TIBC (µg/dL)	P44	507 ± 51	528.5 ± 79.1	532.5 ± 118.9	455.7 ± 38.1
	P51	402.8 ± 53.5	412.5 ± 60.2	388.3 ± 59.6	399 ± 60.4
UIBC (µg/dL)	P44	409.8 ± 75.3	444.7 ± 117	490.8 ± 144.1	370.7 ± 76.2
	P51	336.5 ± 84.9	342.8 ± 99.7	348.5 ± 91.8	310.5 ± 101
Iron (whole blood) (mg/L)	P51	229.7 ± 64.2 ^a^	253.2 ± 65.8 ^ab^	215.7 ± 63.1 ^a^	284.3 ± 49.2 ^b^

**Table 5:** Effects of dietary iron supplementation in the form of iron fortified bread and intramuscular injection on iron concentration (mg·kg^−1^·WM) in organs of post-weanling piglets. Values are expressed as means ± SD; Values in the same row with different superscripts differ significantly *p* < 0.05 (*n* = 6).

Samples		Piglets Group			
		FeMB-G1	FeSB-G2	CB-G3	CB-G4
Intestinal mucosa	P51	25.0 ± 8.1 ^a^	28.0 ± 11.5 ^a^	18.4 ± 4.6 ^b^	27.2 ± 4.4 ^a^
Liver	P51	29.0 ± 7.6 ^b^	30.3 ± 10.1 ^b^	20.0 ± 6.9 ^c^	56.2 ± 24.7 ^a^
Heart	P51	33.0 ± 9.9 ^b^	35.4 ± 8.1 ^b^	26.3 ± 8.4 ^c^	46.0 ± 12.4 ^a^
Spleen	P51	9.4 ± 2.0 ^ab^	10.1 ± 1.2 ^ab^	8.3 ± 1.6 ^b^	10.5 ± 2.4 ^a^
kidney	P51	28.2 ± 8.6 ^ab^	27.3 ± 8.0 ^ab^	24.5 ± 7.3 ^b^	33.0 ± 8.6 ^a^
Feces	P44	130.4 ± 39.7 ^a^	84.2 ± 17.2 ^a^	98.2 ± 31.3 ^a^	119.9 ± 27.3 ^a^
	P51	324.4 ± 129.5 ^b^	168.8 ± 83.7 ^ab^	58.5 ± 18.5 ^a^	139.2 ± 92.4 ^a^
